# Macrophage colony-stimulating factor ameliorates myocardial injury in mice after myocardial infarction by regulating cardiac macrophages through the P2X7R/NLRP3/IL-1β signal pathway

**DOI:** 10.1016/j.heliyon.2023.e20805

**Published:** 2023-10-10

**Authors:** Shu-Juan Zhang, Cong-Xin Huang, Qing-Yan Zhao, He Huang, Jian Zhang

**Affiliations:** a Department of Cardiology, Renmin Hospital of Wuhan University, Wuhan, China; b Cardiovascular Research Insititute, Wuhan University, Wuhan, China; c Hubei Key Laboratory of Cardiology, Wuhan, China

**Keywords:** Myocardial infarction, Macrophage, Inflammation, Apoptosis, Myocardial injury

## Abstract

**Aims:**

To investigate the effects of M-CSF on myocardial injury in mice after MI by regulating different types of cardiac macrophages through the P2X7R/NLRP3/IL-1β signal pathway.

**Methods:**

A total of 60 C57BL/6J WT mice were used, with the Sham Group subjected to ligation without ligation through the LAD, the MI model was prepared by ligation of the LAD in the MC Group and MM Group, with the M-CSF reagent (500 μg/kg/d) being given an intraperitoneal injection for the first 5 days after surgery in the MM Group. All mice were fed in a barrier environment for 1 week. After the study, myocardial tissues were collected and IL-4, IL-6, IL-10, TNF-α, MCP-1, IFN-α, ANP, BNP, β-MHC, Collage I, Collage III, P2X7R, NLRP3, IL-1β, Bax, Caspase 3, C-Casp 3, Bcl-2, M1/2 macrophage, the apoptosis of cardiomyocytes, and the collagen deposition were detected.

**Results:**

The inflammatory response was significantly lower in the MM Group, the cardiomyocyte apoptosis, fibrosis, and hypertrophy were inhibited compared to the MC Group, and the levels of P2X7R, NLRP3, and IL-1β were also statistically lower in the MM Group. Additionally, the expression of M2 macrophages increased in the MM Group while the M1 macrophages statistically decreased compared to the MC Group.

**Conclusion:**

M-CSF can significantly increase the expression of M2 macrophage and reduce the level of M1 macrophage by inhibiting the levels of NLRP3/IL-1β-related proteins, thereby inhibiting inflammation, ameliorating reducing myocardial hypertrophy, apoptosis, and fibrosis, improve myocardial injury in mice after MI.

Myocardial injury after myocardial infarction (MI) significantly increases the incidence of heart failure (HF) and/or ventricular arrhythmias (VAs) [1], with inflammatory response and immune regulatory mechanisms playing an indispensable in the process [2,3]. Studies have confirmed that the excessive inflammatory and immune response aggravate myocardial hypertrophy, apoptosis, and fibrosis [4,5]. Habicht et al. Indicated that initially, the increased inflammatory response is protective in LAD-induced myocardial injury, attenuating the ventricular remodeling [6]. Thus, preventing inflammation has been proven effective in inhibiting myocardial injury after MI, and is an important target for the development of novel strategies to preserve cardiac function.

Purinergic ion-channel receptor 7 (P2X7R), a non-selective cation-gated channel with ATP as a ligand, is mainly highly expressed in immunoregulatory cells including monocytes, macrophages, and lymphocytes [7], and is related to many inflammatory diseases. Many studies have confirmed that inhibition of P2X7R can effectively ameliorate inflammatory diseases [8]. Studies have also found that P2X7R stimulates phosphorylation of signaling molecules (ERK1/2, MAPK, etc.) by activating NOD-like receptors (NLRP3), which then triggers the production of reactive oxygen species, the action of NF-κB, and increases the level of IL-1β to promote inflammatory responses [9]. Cardiac macrophages play a critical role in the injury, remodeling, and repair of inflammatory diseases and act as important immune regulatory cells. Currently, cardiac macrophages are mainly divided into pro-inflammatory M1 macrophages, which secrete pro-inflammatory factors to promote inflammatory responses, and anti-inflammatory M2 macrophages, which are mainly involved in inhibiting inflammatory responses and tissue repair [[10], [11], [12]]. A large number of infiltrating inflammatory factors, chemokines, and different macrophages in the infarct peripheral area are involved in myocardial injury and myocardial tissue remodeling after MI. Therefore, understanding the regulatory effects of different subtypes of cardiac macrophages on inflammatory response and targeting multiple mechanisms are essential to prevent MI injury-induced cardiac dysfunction.

Macrophage colony-stimulating factor (M-CSF, also named Colony-Stimulating Factor-1 or CSF-1), is a specific growth factor dependent on macrophage groliferation and differentiation. M-CSF was considered to be the main driver in the differentation of myeloid precursors toward monocytic lineages and into macrophages, macrophage differentiation requires the interaction between M-ACF and its unique receptor, this interaction triggers a cascade of signaling pathways that promote macrophage differentiation, proliferation, survival and proper functioning [[13], [14], [15]], thus can regulate the polarization and maturation of M1 and M2 macrophages [16,17]. Research has shown that M-CSF is highly expressed in the serum of patients with acute MI and is closely related to the myocardial inflammatory response [18]. However, it is still unclear whether the M-CSF mediates P2X7R/NLRP3/IL-1β signal pathway to ameliorate myocardial injury after MI by regulating cardiac macrophages and its related mechanism. In this study, we prepared a mouse MI model and intraperitoneally injected M-CSF to investigate its effects and mechanisms on myocardial injury by regulating cardiac macrophages through the P2X7R signaling pathway.

## 1 Introduction Methods

1. Animal model preparation: This study was approved by the Animal Studies Subcommittee of Wuhan University School of Medicine and followed the guidelines of the National Institutes of Health for the care and use of laboratory animals. 60 male C57BL/6J wild-type mice (WT) weighing 20–25 g and 8–10 weeks old were selected and raised under the standard laboratory conditions (12:12-h light-dark cycle, temperature 21–25 °C, and 50%–80 % humidity).

All mice were anesthetized with pentobarbital sodium (90 mg/kg), the spleen was resected to clear the entry of splenic and bone marrow derived macrophages in to the spleen. After recovery for 2 weeks under standard conditions, tracheal intubation was performed on the mice while connected to an ECG monitor. The MI model was prepared according to a previously established protocol [19], and all mice were then kept under the same standard conditions.

Sixty WT mice, divided into three groups, were used for the study as follows: Sham group (n = 20), myocardial infarction (MI) group (n = 20), and MI + M-CSF (MM) group (n = 20). Surgery was performed under sterile conditions. A thoracotomy was performed through the third intercostal space and the heart was exposed after cuting the pericardium. In the Sham group, the mice were given a line through the left ventricular coronary artery (LAD) without ligation to prepare the sham operation group. Both the MI and MM groups received a sternotomy followed by ligation of the LAD to prepare the AMI model. MI was confirmed by regional cyanosis and electrocardiographic change (more than two ST segment elevations of 0.1 mV or higher). After this, the M-CSF (500 μg/kg/d) [20] was given an intraperitoneal injection for the first 5 consecutive days in the MM group.The Sham and MI groups were given an intraperitoneal injection of normal saline. All mice were fed in a barrier environment for 1 week.

2. **ELISA:** After the protocol, the myocardial tissues from the 3 groups were stored at −80 °C until analysis. Enzyme-linked immunosorbent assay (ELISA) was used to measure levels of IL-4, IL-6, IL-10, TNF-α, MCP-1, IFN-α, and indicators of myocardial cell hypertrophy and fibrosis (ANP, BNP, β-MHC, Collage I, Collage III).
3. **Western blotting:** Western blotting was performed to detect expression levels of P2X7R, NLRP3, IL-1β, and apoptosis-related proteins (Bax, Casp 3, C-Casp 3, Bcl-2). Ventricular myocardial tissues collected from 3 groups were stored at −80 °C. Primary antibodies against these proteins (rabbit polyclonal anti-proteins antibody used at 1:1000; Abcam) were incubated with the membranes, followed by washing in TBST 3 times and incubation with the secondary antibody for 1 h at 37 °C. The membranes were then imaged using Immun-Star horseradish peroxidase substrate, and the relative expression levels of the proteins were determined using image analysis software (AlphaEase FC, San Leandro, CA, USA).
4. **Immunofluorescence staining:** Immunofluorescence staining was utilized to analyze the levels of different polarization subtypes of M1 and M2 macrophages in myocardial tissue from the 3 groups. At the end of the study, ventricular tissues were collected, fixed with 4 % formaldehyde diluted in warm PBS for 15 min at room temperatur,e and washed 3 times in PBS. The tissues were then blocked using 5 % normal serum and 0.3 % Triton X-100 in PBS for 1 h at room temperature. Subsequently, the tissues were incubated overnight at 4 °C with an antibody against iNOS and Arg-1 (1:100; ab 10092; Abcam). After being rinsed 3 times with PBS for 5 min each, the specimens were incubated in a 1:50 dilution of CY3-labeled goat anti-rabbit secondary antibody (1:50; AS-1109; Aspen) for 1 h at room temperature in the dark and washed 3 times in PBS again. PI counterstaining was performed to stain the nuclei. Finally, the coverslips were mounted, and images were viewed using an Olympus IX51 fluorescence microscope and QIMAGING Micropublisher.
5. **Others:** TUNEL assay was conducted to evaluate myocardial cell apoptosis, while collagen deposition in myocardial tissue was assessed through PSR staining.
6. **Statistical analysis:** Data were presented as mean ± standard deviation. Paired *t*-test was used to compare continuous variables at the end of the study with those at baseline. One-way analysis of variance with Tukey's test was employed to compare the means of continuous variables among multiple groups. All statistical tests were two-tailed, and a P-value <0.05 was considered statistically significant.

## 2 Results

1. **Base condition:** During the experiment, 1 mouse in the Sham Group and 9 mice in the MC Group died, while 5 mice died in the MM Group. The remaining mice completed the experiment without complications. There were no significant differences in body weight and basal heart rate among the 3 groups before the study (Fig. 1).

**Fig. 1:**
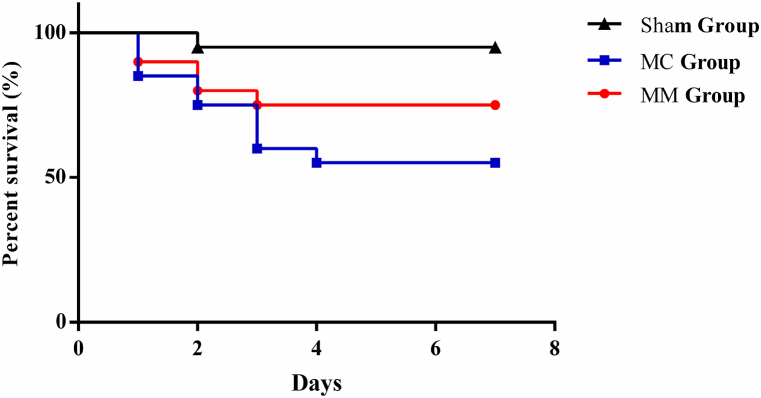
Cumulative survival rate of the 3 groups.
2. **Inflammation factors:** As shown in Table 1, compared with the Sham Group, the levels of pro-inflammatory factors, such as IL-6, TNF-α, and MCP-1, in the myocardial tissues of the mice in the MC and MM Group were significantly higher (all *P* < 0.05). However, the levels of these factors were lower in the MM Group than in the MC Group (all *P* < 0.05). Compared with the MC Group, the levels of anti-inflammatory cytokines, such as IL-4, IL-10, and IFN-α, in the myocardial tissue of the MM Group mice significantly increased, and the difference was statistically significant (all *P* < 0.05).

**Table 1:** Comparison of the inflammatory factors in myocardial tissue of the 3 groups. *: vs Sham Group, *P*＜0.05; #: vs MC Group, P＜0.05.

References	Group		
	Sham Group （n = 19）	MC Group （n = 11）	MM Group （n = 15）
IL-6 (pg/ml)	176.50 ± 7.14	235.27 ± 6.34*	205.27 ± 9.92*^#^
TNF-α (pg/ml)	51.45 ± 2.06	88.45 ± 6.31*	78.52 ± 3.38*^#^
MCP-1 (pg/ml)	92.80 ± 12.93	194.75 ± 26.28*	169.04 ± 24.53*^#^
IL-4 (pg/ml)	51.80 ± 4.23	41.18 ± 5.97*	87.32 ± 12.77*^#^
IL-10 (pg/ml)	78.27 ± 3.60	107.45 ± 6.05*	276.99 ± 7.46*^#^
IFN-α (pg/ml)	77.71 ± 3.99	13.49 ± 1.06*	21.36 ± 8.47*^#^
3. **Myocardial cell apoptosis:** The apoptosis rate of myocardial cells in the MC Group and MM Group was significantly higher than that in the Sham Group, while it was lower in the MM Group than in the MC Group (all *P* < 0.05) (Fig. 2 A, B). When compared with the Sham Group, the levels of apoptotic proteins (Bax, Caspase 3, and C-Casp 3) in myocardial tissue in the MC Group and MM Group were significantly higher (all *P* < 0.05), and the levels of anti-apoptotic protein Bcl-2 in MC Group and MM Group significantly decreased (all *P* < 0.05). When compared with the MC Group, the expression of Bcl-2 in the MM Group was significantly higher, while the levels of Bax, Caspase 3, and C-Casp 3 were significantly decreased (all *P* < 0.05) (Fig. 2 C, D).

**Fig. 2:**
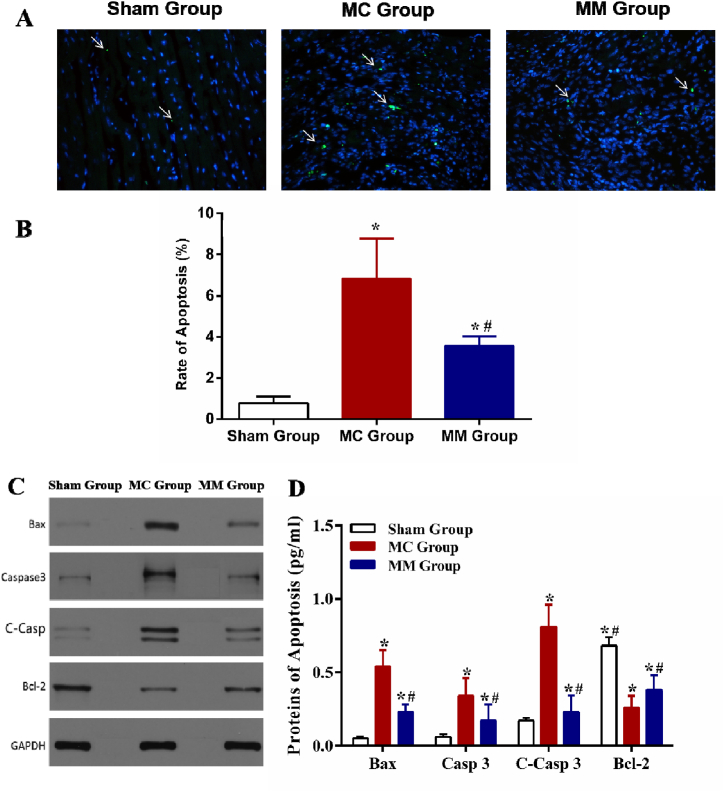
Comparison of the apoptosis and related proteins in myocardial tissues of the 3 groups. (A) The results of TUNEL staining. (B) Statistical chart of apoptosis rate of cardiomyocytes in 3 groups. (C) Levels of apoptosis-related proteins in 3 groups. (D) Statistical chart of the levels of apoptosis-related proteins in 3 groups. *: vs Sham Group, *P*＜0.05; #: vs MC Group, *P*＜0.05。.
4. **Myocardial hypertrophy, fibrosis, and collagen deposition:** As shown in Fig. 3, when compared with the Sham Group, the levels of ANP, BNP, and β-MHC in the MC Group and MM Group significantly increased, while they were significantly lower in the MM Group than in the MC Group (all *P* < 0.05) (Fig. 3 A). Compared with the Sham Group, the fibrosis-related markers (Collage I and Collage III) in the MC Group and MM Group were significantly higher after MI, while the expressions of Collage I and Collage III in the MC Group were statistically higher than those in the MM Group (all *P* < 0.05) (Fig. 3 B).

**Fig. 3:**
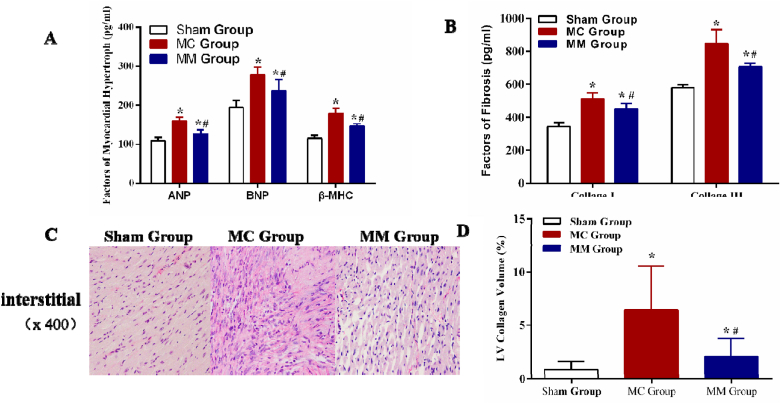
Comparison of the myocardial hypertrophy, fibrosis, and collagen deposition in 3 groups. (A) Comparation of the myocardial hypertrophy in 3 groups; (B) Comparation of the myocardial fibrosis in 3 groups; (C) and (D) Comparation of the myocardial collagen deposition in 3 groups. *: vs Sham Group, *P*＜0.05; #: vs MC Group, *P*＜0.05.

The PSR results showed that when compared with the Sham Group, there was a large amount of collagen fiber infiltration in the peripheral area of MI in the MC Group and the MM Group (all *P* < 0.05). The collagen deposition volume fraction in the myocardial interstitium in the MM Group was lower than that in the MC Group (*P* < 0.05) (Fig. 3 C, D).

5. **Results of P2X7R, NLRP3, and IL-1β proteins in 3 groups:**Fig. 4 indicates that the expressions of P2X7R, NLRP3, and IL-1β in myocardial tissue were significantly higher in both the MC Group and MM Group than in the Sham Group (all *P* < 0.05). However, the expressions of these proteins were lower in the MM Group than in the MC Group (all *P* < 0.05) (Fig. 4 A–E).

**Fig. 4:**
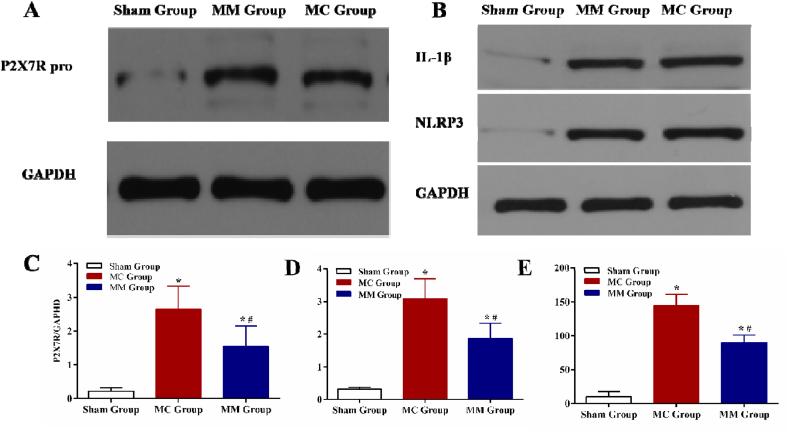
The results of P2X7R/NLRP3/IL-1β in 3 groups. (A) and (C) Comparison of the P2X7R proteins in 3 groups; (B), (D), and (E) Comparasion of the NLRP3/IL-1β proteins in 3 groups. *: vs Sham Group, *P*＜0.05; #: vs MC Group, *P*＜0.05.
6. **Results of M1, and M2 macrophages:** As shown in Fig. 5, the expressions of M1 and M2 macrophages in myocardial tissues were higher in the MC Group and MM Group than in the Sham Group (all *P* < 0.05). The level of M1 macrophage was significantly lower in the MM Group (*P* < 0.05), while the levels of M2 macrophage were higher in the MM Group (*P* < 0.05) (Fig. 5 A, B).

**Fig. 5:**
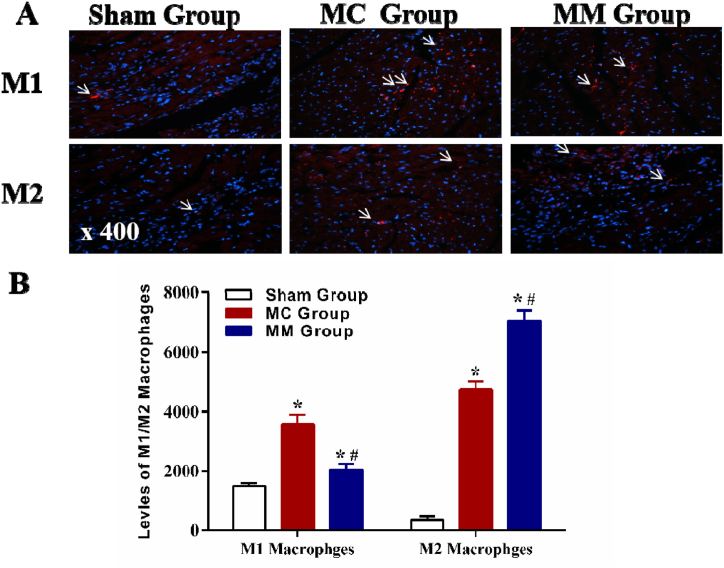
Comparison of the levels of M1 and M2 macrophages in 3 groups. (A) Results of the immunofluorescence staining; （B）Statistical chart of M1 and M2 Macrophages in 3 groups. *: vs Sham Group, *P*＜0.05; #: vs MC Group, *P*＜0.05.

## 3 Discussion

The pathophysiological processes of myocardial injury, HF, and VAs after MI are complex and involve various inflammatory factors, chemokines, and signaling pathways [21]. Numerous studies have demonstrated that acute injury, apoptosis, necrosis, and intercellular fibrosis of cardiomyocytes can prompt an inflammatory response which triggers the systematic innate immune response to phagocytose and clear apoptotic, necrotic cells and debris in the MI area after MI [[21], [22], [23]]. Early research has revealed that a mild inflammatory and immune response can decrease the structural remodeling of cardiomyocytes, promote the scar tissue formation, maintain homeostasis in the MI area, and enhance ischemic myocardial repair. However, excessive inflammatory and immune responses can induce apoptosis, hypertrophy, fibrosis, and necrosis of cardiomyocytes in non-MI regions, aggravating myocardial pathological remodeling and dysfunction after MI [[24], [25], [26]]. After the occurrence of AMI, the earlier the intervention to reduce myocardial inflammatory response, the better the improvement of myocardial injury, and the greater the benefit. Therefore, regulating the inflammatory and immune responses has become an important means of preventing and treating ventricular remodeling, HF, and VAs after MI at present and in the future. Kim et al. Studied whether activate the immune inflammatory system before the occurrence of reperfusion injury could regulate the inflammatory response and structure remodeling after myocardial infarction in reperfusion mice, and found that postconditioning after reperfusiond myocardial infarction is associated with modulated infallation, less apoptosis, and better left ventricular function [27]. However, since the mechanisms of inflammatory response in the pathophysiological process of MI have not been fully elucidated and the individual differences of MI patients are significant, complications after MI are more complicated, necessitating considerable basic and clinical research to study its phenomenon and mechanism. Therefore, in this study, a mouse model of acute MI was prepared, and the expression levels of different polarized subtypes of macrophages in the heart were regulated by intraperitoneal injection of M-CSF reagent to explore their mechanism of action o the inflammatory response and myocardial cell injury after MI.

Cardiac macrophages are important immune-regulatory cells with dual roles in the inflammatory response after MI. They can ameliorate the injury and remodeling of myocardial cells after MI by releasing pro-inflammatory cytokines (such as IL-6, TNF-α, etc.) while also secreting anti-inflammatory cytokines (such as IL-4, IL-10, etc.) to inhibit the inflammatory response and promote myocardial repair after MI [28,29]. M1 macrophages highly express iNOS and secrete pro-inflammatory factors such as IL-6, TNF-α, etc. to present antigens, and phagocyte debris, and promote inflammatory responses. On the other hand, M2 macrophages highly express Arg-1, mainly promote cell differentiation and proliferation, inhibit the inflammatory response, and participate in tissue repair. Under certain cytokines and micro-environmental conditions, they can transform into each other [[10], [11], [12]]. As is well known, M-CSF is very important for the polarization and maturation of cardiac macrophages and is also a sensitive indicator of inflammatory diseases [16,17,30]. In addition, M-CSF is very important for the polarization and maturation of cardiac macrophages and is also a sensitive indicator of inflammatory diseases [16,17,30]. Although the impact of the macrophages and inflammation on MI induced myocardial injury has been extensively investigated, but not finally elucidated. Kim et al. reported that early and significantly higher upregulation of macrophage chemoattratant MCP-1 after MI, being significantly stronger after postcondition, possibly indicating a stronger signal for macrophage differentiation or activation in the ischemic myocardium, and myocardial injury was significantly reduced after the expression of M1 macrophages was lower [27]. George et al. Studied the effects of CB2 on myocardial injuries, such as inflammation, apoptosis, and fibrosis after myocardial ischemia-reperfusion injuries in mice, and discussed ots related mechanisms, they pointed that cardiac macrophages played an important role in the inflammatory response and myocardial repair after myocardial ischemia-reperfusion injuries [31,32]. Based on the above research content, we prepared an AMI model and administered an M-CSF reagent through intraperitoneal injection to investigate the hypothesis that M-CSF may prevent deleterious consequences and its mechanisms in myocardial tissues after AMI in mice. Our results showed that both of M1 and M2 macrophages increased significantly after AMI, after intraperitoneal of M-CSF, M2 macrophages in the MM Group were significantly more than those in MC Group, and the expression level of M2 macrophages in the MM Group was also higher than that of M1 macrophages. This result indicated that M-CSF could inhibit the expression of M1 macrophages and up-regulated the level of M2 macrophages.

We further investigated the effects of M-CSF on inflammatory response and myocardial injury after AMI in mice, and found that after AMI, the pro-inflammatory cytokines (such as IL-6, TNF-α, and MCP-1) in the myocardial tissue significantly increased while the anti-inflammatory cytokines (IL-4, IL-10, and IFN) were significantly decreased. However, the pro-inflammatory factors decreased and the anti-inflammatory cytokines were higher after intraperitoneal of M-CSF.

The results confirmed that after the conduction of AMI in mice, injection of M-CSF intraperitoneally can effectively upregulate the expression of M2 macrophages in the heart and inhibit the inflammatory response. In addition, we also observed myocardial injuries in mice after AMI, and found that, the myocardial apoptosis, cardiomyocyte hypertrophy, apoptosis, fibrosis deteriorated after AMI, the results of Sirius red staining showed that the collagen deposition in the myocardial tissue in the MC Group significantly increased than in the Sham Group, however, M-CSF could ameliorate these changes and improved myocardial damage. The results above indicated that M-CSF played an important role in myocardial tissue repair, but its mechanism is not fully understood. Above findings suggested thta cardiac macrophages play an important role in myocardial inflammatory response and myocardial injuries after AMI.

P2X7R is widely distributed in a variety of immune regulatory cells, such as lymphocytes and macrophages, and has a very important relationship with a variety of inflammatory diseases [33]. A previous study has found that the P2X7R signaling pathway promotes the secretion of IL-1β by activating the NOD-like receptor (NLRP3), which in turn promotes the polarization and maturation of M1-type macrophages. Drug blocking of P2X7R could significantly reduce the inflammatory response and sympathetic nerves after MI [9], but its mechanism still needs to be further studied. Therefore, we detected the P2X7R/NLRP3/IL-1β signaling pathway protein in the myocardial tissues of the 3 groups to explore the mechanism by which M-CSF improves myocardial injury after AMI in mice. The results showed that the expression levels of P2X7R, NLRP3, and IL-1β in the myocardial tissue of mice after AMI were significantly increased compared with those in tha Sham Group, while the expression levels of above proteins in the MM Group decreased after intraperitoneal injection of M-CSF, and the difference was statistically significant. Previous studies have found that the cardiac macrophages, as one of the important immune regulatory cells, are of great significance in the occurrence and development of MI [34], the NLRP3 inflammasome plays an important role in myocardial injury after MI [35], and IL-1β can promote the polarization and maturation of M1 macrophages and inflammatory response by activating downstream signaling cascade responses, while M2 macrophages suppress the inflammatory response by secreting anti-inflammatory cytokines, promote tissue phagocytosis, repair, and ameliorate myocardial injury after MI [36,37]. In this study, AMI mice were given intraperitoneal injection of M-CSF, the expression levels of M2 macrophages significantly increased, while the levels of M1 macrophages statistically decreased, and myocardial inflammation, apoptosis, hypertrophy, and other myocardial injuries were significantly improved, the expressions of P2X7R\NLRP3 signaling pathway proteins decreased. We conclude that M-CSF may regulate cardiac macrophages by mediating the P2X7R\NLRP3 signaling pathway to ameliorate myocardial injuries after AMI.

The polarization, maturation, and migration of cardiac macrophages are very complex, and many signaling pathways are involved, which have not been fully elucidated. Although there are lots of basic and clinical studies on the role of cardiac macrophages in inflammatory response and myocardial injury after AMI, more experiments are still needed to explore the specific mechanism. The results of our study suggested that M-CSF could inhibit the inflammatory response, cardiomyocyte hypertrophy, apoptosis, fibrosis, etc. and myocardial injury after MI by increasing the polarization and maturation of M2 macrophages and inhibiting the inflammatory effect of M1 macrophages, P2X7R/NLRP3/IL-1β signaling pathway played an essential role in this process. The results of our study suggest that early intervation after AMI can improve myocardial injuries effectively by increasing the immune activity of M2 macrophages and promoting their role in tissue repair.

## 4 Ethic statement

This study has been approved by the Laboratory Animal Welfare & Ethics Committee of Renmin Hospital of Wuhan University (No. 20191211), and all authors complied with all relevant ethical regulations.

## Author contributions

Shujuan Zhang and Qingyan Zhao provided the idea. Shujuan Zhang designed the outline, reviewed the literature, drafted the manuscript, and designed the figures. Congxin Huang and Qingyan Zhao provided the funding. Qingyan Zhao, He Huang, and Congxin Huang guide the writing and revision of the article. Shujuan Zhang and Jian Zhang were involved in refining and polishing the manuscript. All authors contributes to the article and approved the submitted version.

## Funding

This study was funded by the 10.13039/501100001809National Natural Science Foundation of China (No. 81970277), the special fund for Technology Innovation of Hubei Province (Major Project) (No. 2016 ACA 153), and the Central Universities’ Basic Research Funding Special Fund (No. 2042022kf1111).

## Publisher's note

The claims expressed in this article are solely those of the authors and do not represent those of their affiliated organizations or the publisher, the editors, and the reviewers. Any products evaluated in this article or claims made by manufacturers are not guaranteed or endorsed by the publisher.

## Declaration of competing interest

The authors declare that they have no known competing financial interests or personal relationships that could have appeared to influence the work reported in this paper.
